# A Unique Physical Therapy Approach in a Complex Case of Pott's Fracture: A Case Report

**DOI:** 10.7759/cureus.36847

**Published:** 2023-03-29

**Authors:** Anushka Raipure, Pratik Phansopkar, Shivani R Uttamchandani

**Affiliations:** 1 Musculoskeletal Physiotherapy, Ravi Nair Physiotherapy College, Datta Meghe Institute of Medical Sciences, Wardha, IND

**Keywords:** bimalleolar ankle fracture, physiotherapy, rehabilitation, pott’s fracture, ankle fractures

## Abstract

The ankle joint is a complex joint that bears the human body's weight throughout daily activities. Unimalleolar, bimalleolar, and trimalleolar fractures are the three subgroups that make up the category of ankle fractures. Determining the risk-benefit tradeoffs between non-operative and surgical therapy still requires a thorough initial examination of the fracture pattern, soft tissue condition, and patient characteristics. Ankle fractures that are stable and well-aligned respond well to conservative therapy. Open reduction and internal fixation is the current gold standard of treatment for displaced and unstable fracture patterns, with historical data revealing good to outstanding outcomes for the majority of these patients. We present a case of a 19-year-old female sustaining a Pott’s fracture following a road traffic accident. The overall treatment technique used included the overload principle, task-specific training, and an impairment-based strategy. The patient's impairments with regard to the range of motion, strength, edema, discomfort, wound healing, and functional limitations were handled by the therapist using exercises, manual therapy, and compressive cryotherapy. A clinically significant rehabilitation protocol for treating Pott's fracture is established in this case report.

## Introduction

The complex ankle joint supports the human body's weight throughout regular activities [1]. The compound articulations of the talocrural, distal tibiofibular, and subtalar joints make up the ankle complex. Although the architecture and functions of these three joints are frequently described individually, they are interconnected in a complex way that prevents them from being separated. The intersection of the tibia and fibula makes up the tibiofibular joint. This joint is categorized as a plane synovial joint up close because gliding occurs between the articulating surfaces. The talocrural joint is a stabilized uniaxial modified hinge synovial joint. It comprises the distal articular surfaces of the tibia and fibula and the proximal articular surface of the talus (trochlea). Dorsiflexion and plantar flexion make up most motion at the talocrural joint. The bicondylar articulation between the talus and calcaneus makes up the subtalar joint. Due to its tri-planar mobility, the arthrokinematics of the subtalar joint can be quite complex [2]. Ankle fractures are a common fracture of the lower extremities, occurring at a rate of 187 per 100,000 individuals each year [1]. Ankle fractures are divided into unimalleolar, bimalleolar, and trimalleolar fractures [1]. Bimalleolar fractures, often called Pott's fractures, are more common in women and older persons. The most common mechanism of injury that damages this ligament is foot eversion or an external rotation force due to the intense medial stresses these forces produce [3]. The fracture pattern, soft tissue condition, and patient characteristics must be thoroughly examined to compare the risks and benefits of non-operative versus surgical therapy [4]. Ankle fractures that are stable and well-aligned respond well to conservative treatment. Open reduction and internal fixation (ORIF) is the current gold standard of treatment for displaced and unstable fracture patterns, with historical data revealing good to outstanding outcomes for most of these patients [5].

The Danis-Weber and Arbeitsgemeinschaft fuer Osteosynthesefragen classifications are straightforward for everyday usage and clinical application and are based on radiographic data. The Weber classification system is often used to classify ankle fractures [6]. Based on the severity of the fibula's fracture and the degree of syndesmosis disruption, Harper aims to classify this fracture as one of three types: A, B, or C. The syndesmosis and the tibiofibular ligaments are unaffected by the Weber A fracture. The fibula has a transverse fracture at or below the syndesmosis level. Fibula fractures, known as Weber B fractures, begin at the level of the syndesmosis and rupture some of the tibiofibular ligament. Weber C fractures are those in which the diaphysis of the fibula is shattered, and the syndesmosis is disturbed. Weber A fractures require conservative treatment, but Weber C fractures necessitate surgery, open reduction, and internal fixation [7]. The majority of ankle fractures are Lauge-Hansen supination-external rotation fractures, also known as Weber B or Orthopaedic Trauma Association type B [8]. The integrity of the syndesmosis and patient characteristics like age and comorbidities will affect how these fractures are managed. While more unstable injuries are typically treated surgically with ORIF utilizing various techniques, such as plates and screws or tightrope, more stable injuries are frequently treated conservatively with close contact casts or braces [7]. We present a case of a 19-year-old patient who sustained a bimalleolar ankle fracture following a fall from a road traffic accident with a twisting mechanism. This report aims to provide a thorough assessment and physiotherapy management protocol. The patient was evaluated using a model from the International Classification of Functioning, Disability, and Health (ICF). This report would help to provide evidence-based treatment protocol and help to improve the quality of treatment.

## Case presentation

A 19-year-old female, a student by occupation, was brought to casualty by her father with complaints of pain and swelling over the right ankle. The patient gave an alleged history of a road traffic accident on March 21, 2022, sustaining an injury to the right ankle, after which the complaints started, and the patient could not bear weight on her right ankle. Immediately after the injury, the patient developed pain. The pain was associated with swelling that was constant throughout and not radiating. The pain was aggravated with movement and was relieved with rest and medications. The patient also sustained injury over the hand and left eyebrow.

The patient underwent an ORIF on March 29, 2022. Antibiotics and analgesics were administered immediately after surgery. The patient received a below-knee slab for the first four weeks. The patient was diligent with cryotherapy and elevation and consented to participate in early physiotherapy.

The therapist took a well-informed consent before the examination. The patient had significant pain intensity during the initial assessment. Based on the numeric pain rating scale (NPRS), she felt 7/10 average discomfort on the right ankle. She reported pain 9/10 on NPRS when standing and walking particularly ankle inversion and eversion. The patient reported cryotherapy, elevation, and non-weight bearing were easing influences. She admitted that she needed help with daily activities, such as showering. She walked around with the help of a walker. The patient's primary complaints were discomfort, difficulties doing activities of daily living (ADLs), and the inability to attend school. The patient belonged to the upper middle class based on the Kuppuswamy scale. The patient lives in a well-furnished house with a western-style toilet and is well-supported and motivated by her parents. Pre-rehabilitation assessment was done using the ICF model. Tabled 1-2 show the diagnostic evaluation of the patient.

**Table 1:** Assessment of the patient in the body structure and function category NPRS: numeric pain rating scale

Category	Test/outcome used	Left	Right
Pain	NPRS	On rest 7/10	During activity 9/10
Swelling	Figure 8 girth measurement	57 cm	59 cm
Active range of motion	Goniometry	Dorsiflexion 0-10, plantarflexion 0-55, inversion 0-33, eversion 0-16	Not assessed
Muscle strength	Manual muscle testing	Dorsiflexors 5/5, plantar flexors 5/5, invertors 5/5, evertors 5/5	Not assessed

**Table 2:** Assessment of the functional activity and participation restriction category FAAM: foot and ankle ability measure, LEFS: lower extremity function scale

Category	Measure used	Results
Fall risk	Berg Balance Scale	19/56
Lower extremity functional status	FAAM, LEFS	17/84, 9/80
Ambulation	Patient report and observation	The patient reported being able to ambulate in the ward using a walker
Attending school	Patient report	Without an assistive device, the patient reported being unable to ambulate in the community, which was necessary for going to school

The overall treatment technique included the overload principle, task-specific training, and an impairment-based strategy. The therapist handled the patient's impairments regarding the range of motion, strength, edema, discomfort, wound healing, and functional limitations using exercises, manual therapy, and compressive cryotherapy. Coordinating treatment with the patient's surgeon enabled the establishment of appropriate care that best suit the patient's objectives and circumstances. The phase-wise goals and the interventions are listed in Tables 3-6.

**Table 3:** Weeks 1-4 goals and interventions NPRS: numerical pain rating scale, FAAM-ADL: foot and ankle ability measure-activities of daily living, LEFS: lower extremity function scale

Problem	Goals	Interventions
Pain in the medial and lateral aspects of the right ankle	Reduce patient-reported pain on rest to 5/10 and on activity to 8/10 NPRS in the right ankle.	The patient executed therapeutic procedures at a pain level no more than the average to prevent symptoms from worsening. Cryotherapy: 10 minutes after therapy.
Swelling in right ankle and foot	Reduce swelling to a 59 cm measurement of the right ankle with the figure 8 method.	The patient is informed about the correct use and advantages of using a bandage, foot elevation, general mobility, and compression to help reduce edema—Cryotherapy finger-toe movements within the cast 10 repetitions for three sets.
Decrease in proper leg range of motion	Maintain range of motion of the knee and hip joint range of motion.	The patient performed the following exercises 10 times in three sets: finger-toe movements, straight leg raising, hip abduction-adduction, and knee flexion-extension.
Decreased right ankle muscular strength	Maintain the integrity of right leg musculature.	The patient performed the following exercises 10 times in three sets: static quadriceps, static back, static abdominals, static glutes, and vastus medialis oblique strengthening.
Increased fall risk	Improve the patient's status from high fall risk to medium fall risk on the Berg Balance Scale.	The patient receives instructions on how to use mobility aids safely.
Limited functional status	Improved function with a FAAM-ADL score of 25/84. Improve functions with a LEFS score of 20/80.	The patient is encouraged to avoid sedentary behavior and is advised moderate exercise to promote healing and overall health gains.
Limp during ambulation	Increase tolerance to ambulation.	For the first two weeks, the surgeon prescribed no weight bearing. Toe-touch weight bearing can begin after two weeks. The patient utilized a walker for the first two weeks, followed by controlled ankle motion boots.
Unable to attend school	Make the patient independent to attend school again.	The therapist urged the patient to focus on the rehabilitation program for a quick return to school.

**Table 4:** Weeks 4-6 plan of care NPRS: numeric pain rating scale, FAAM-ADL: foot and ankle ability measure-activities of daily living, LEFS: lower extremity function scale, CAM: controlled ankle motion

Problem	Goals	Interventions
Pain in the medial and lateral aspects of the right ankle	Reduce patient-reported pain on rest to 3/10 NPRS and on activity to 6/10 NPRS.	Soft tissue massage relieves discomfort in the medial, lateral, and anterior ankle joints and the anterior foot. (3 minutes of manual light intensity), cryotherapy.
Swelling in right ankle and foot	Reduce the right ankle's swelling to a 58 cm measurement with the figure 8 method.	Retrograde soft tissue massage: 3 minutes, toe curls: 10 repetitions for three sets, and cryotherapy.
Decreased suitable ankle range of motion	Increase active range of motion of right ankle dorsiflexion to neutral, plantarflexion to 0-35, inversion to 0-30, and eversion to 0-13.	The patient performed the following ten times in three sets, along with phase 1 exercises: active ankle pumps, active inversion and eversion, seated heel slides for ankle dorsiflexion, and toe stretching.
Decreased right ankle muscular strength	Improve the strength of ankle musculature to 3/5 on manual muscle testing.	The patient performed the following exercises 10 times in three sets with a 10-second hold. Initiate isotonic exercises for ankle dorsiflexors, plantar flexors, invertors and evertors, prone hip extension, and clamshells.
Increased fall risk	Same as phase 1.	Same as phase 1.
Limited functional status	Improved function with a FAAM-ADL subscale score of 33/84 and a LEFS score of 31/80.	The exercises, as mentioned above, supported the improvement of functional status.
Limp during ambulation	Increase tolerance to ambulation.	Starting around week four, patients will begin partial weight bearing, with 25% of one's body weight and adding 25% until one is fully weight bearing in a boot; the patient is gradually weaned off CAM boots.

**Table 5:** Weeks 6-8 plan of care NPRS: numeric pain rating scale, BAPS: biomechanical ankle platform system, SLB: single-limb balance, FAAM-ADL: foot and ankle ability measure-activities of daily living subscale, LEFS: lower extremity function scale

Problem	Goals	Interventions
Pain in the medial and lateral aspects of the right ankle	Reduce patient-reported pain on rest to 1/10 and activity to 4/10 NPRS in the right ankle.	Soft tissue massage: 3 minutes with increased intensity and cryotherapy.
Swelling in right ankle and foot	Reduce swelling to a 57 cm measurement of the right ankle with the figure 8 method.	Soft tissue massage: 3 minutes of increased manual intensity, ankle pumps, toe curls: 30 repetitions for two sets and cryotherapy.
Decreased suitable ankle range of motion	Increase active range of motion of right ankle dorsiflexion to 0-10, plantarflexion to 0-50, inversion to 0-35, and eversion to 0-15.	Stretching exercises (standing quad stretch, standing hamstrings stretch, Thomas hip flexor stretch, piriformis stretch, toe stretch). Once weaned from the boot, perform standing gastrocnemius and soleus stretching, talocrural joint glides (anteroposterior and posteroanterior): begin with grade 1/II x30 seconds, active ankle pumps and active inversion and eversion: progressed to 2x30 repetitions, half foam roll inversion and eversion: seated with foot on half foam roll 30 repetitions, active calf stretch with assistance or supervision, towel sweeps: 30 repetitions for two sets.
Decreased right ankle muscular strength	Able to tolerate medium resistance TheraBand of dorsiflexion, inversion, and eversion and a seated single-leg heel raise.	TheraBand resists ankle ranges and seated heel raises: begin with a light resistance band for three sets with 10 repetitions. Seated heel raises, isotonic exercises, clamshells, and prone hip extensions: 15 repetitions for three sets.
Increased fall risk	Improving Berg Balance Scale score.	Wean off the boot and fit with an air cast in a regular shoe. When switching to ordinary shoes, ambulate inside the home before moving outside; boost weight-bearing to the patient's tolerance; do daily stretching in advance. Here, we are advancing from ankle isometrics to open-chain isotonic, exercises with a closed chain (weight machines, weight shifts, seated BAPS), exercises for improving proprioception (SLB, diagonal doing, and foot intrinsic strengthening), metatarsal mobilization: begin with manual light intensity for 30 seconds, passive toe flexion and extension: begin with x30 seconds, weight shifting side to side with rotation: begin for 30 seconds, progress to 3x30 seconds, weight moving forward and backward: weight shifting in walk stance on each side with an emphasis on heel-to-toe, progressed to treadmill training.
Limited functional status	Improved function with a FAAM-ADL subscale score of 48/80 and a LEFS score of 43/80.	Ergonomic advice, weight shifting side to side with rotation.
Limp during ambulation	Able to tolerate ambulation at home.	At week seven, the patient was advised weight bearing as tolerated.
Unable to attend school	They are not anticipated to tolerate community ambulation at this time.	The patient was advised to increase weight-bearing and walking time as tolerated by the patient.

**Table 6:** Weeks 8-12 plan of care NPRS: numerical pain rating scale, FAAM-ADL: foot and ankle ability measure-activities of daily living subscale, LEFS: lower extremity function scale, BAPS: biomechanical ankle platform system

Problem	Goals	Interventions
Pain in the medial and lateral aspects of the right ankle	Reduce patient-reported pain on rest to no discomfort and pain on activity to 1/10 NPRS in the right ankle.	Soft tissue massage: 5 minutes with increased intensity, and cryotherapy home exercise program: We suggested the patient use a cold pack or immerse the right ankle in cold water for 10 minutes, at least three times a day.
Swelling in right ankle and foot	To maintain the achieved result.	The patient was administered soft tissue massage, ankle pumps, toe curls: 30 repetitions for three sets and cryotherapy. Home exercise program: advise the patient to perform ankle pumps and toe curls daily.
Decreased suitable ankle range of motion	Maintain the achieved range of motion.	Maintain toe stretches and active range of motion exercises as necessary. Once talocrural joint mobility returns to normal, standing gastrocnemius and soleus stretching is performed. Talocrural joint glides (anteroposterior and posteroanterior): begin with Grade 1/II x30 seconds. Active ankle pumps, towel sweeps, and active inversion and eversion: progressed to 3x30 repetitions. BAPS board: started with half a foam roll and worked up to BAPS board. Actively assisted calf: advanced to elevating leg with straight knee 3x30 seconds. Home exercise program: the patient is instructed to perform these exercises daily.
Decreased right ankle muscular strength	Increase dorsiflexor, invertor, and evertor muscle strength to 4/5. Able to perform a standing single-leg heel raise (3/5).	Leg press: to encourage ankle mobility. It began with an emphasis on equal weight bearing and then progressed by adding weights. The patient then progressed with adding upper extremity-assisted mini squats at Week 12. Seated heel raises: progressed with the addition of ankle weights. Continue previous exercises with a 15-second hold. Home exercise program: continuing the same protocol and progressing according to the patient’s performance.
Increased fall risk	We are improving Berg Balance Sore to 51/56, indicating a low risk of falls.	Progression was made according to the patient’s tolerance, and a suitable home program was suggested.
Limited functional status	Improved function with a FAAM-ADL subscale score of 48/80 and a LEFS score of 43/80.	The patient was given information on the value of following a home program to reduce impairments and restrictions and enhance overall functional status.
Limp during ambulation	Able to tolerate ambulation.	After Week 12, full weight bearing can begin, and the same interventions are continued.
Unable to attend school	Able to tolerate community ambulation for 30 minutes.	Interventions are to be continued as outlined above.

Tables 7-8 show the pre- and post-rehabilitation outcome measures.

**Table 7:** Outcome measures for pain, skin integrity, balance, foot and ankle measure, lower extremity scale, ambulation, and return to work pre- and post-intervention

Measure	Pre-rehabilitation	Post-rehabilitation
NPRS on rest	7/10	1/10
On activity	9/10	4/10
Skin incisions	Lateral surgical incision presented with a small open wound and mild scabbing. The medial surgical incision is shown with mild scabbing and skin dryness.	Increased healing without scabbing or skin dryness, with signs of better skin integrity and no noticeable scarring.
Berg Balance Scale	19/56	49/56
Foot and ankle ability measure	17/84	61/84
Lower extremity function scale	9/80	58/80
Ambulation ability	The patient reported being able to ambulate in the ward using an assistive device.	Ambulation ability is increased by 25 minutes without aids in the house. The severity of limping was reduced.
The patient reported a return to school.	Without an assistive device, the patient reported being unable to attend school.	With a 30-minute improvement in tolerance, the patient could now resume school.

**Table 8:** Outcome measures for goniometry, girth measurement, and manual muscle testing pre- and post-intervention Unit for goniometry values in degree

Measure	Pre-rehabilitation		Post-rehabilitation	
Goniometry	Left	Right	Left	Right
Plantarflexion	0-55	Not assessed	0-55	0-50
Dorsiflexion	0-10	Not assessed	0-10	0-10
Inversion	0-33	Not assessed	0-33	0-30
Eversion	0-16	Not assessed	0-16	0-12
MMT	Left	Right	Left	Right
Plantarflexors	5/5	Not assessed	5/5	3/5
Dorsiflexors	5/5	Not assessed	5/5	4/5
Invertors	5/5	Not assessed	5/5	4/5
Evertors	5/5	Not assessed	5/5	4/5
Figure 8 girth	Left	Right	Left	Right
(in cm)	57 cm	59 cm	57cm	57 cm

## Discussion

Following an ORIF for a right bimalleolar fracture, the patient had the usual impairments and functional limits for which physical rehabilitation was given. After receiving physical therapy, the patient displayed gains in active status and all deficits. The patient made progress in achieving her objective of going back to school. The overload principle was applied to encourage advancement and workload adjustments. These adjustments enabled the patient to handle more activity and demand while advancing toward short- and long-term objectives. Additionally, the protocol also included education regarding precautions, optimizing healing, and letting the patient know what to expect from their recovery.

There is little evidence to guide care for people with surgically repaired ankles. Following surgery, these individuals commonly have a restricted range of motion, loss of function, and post-surgical immobility. Three sessions of impairment-based manual therapy did not result in more significant improvements in mobility, gait, or balance for patients undergoing an ORIF for an ankle or hindfoot fracture when compared to a control group receiving soft-tissue mobilization and proximal tibiofibular joint mobilization, according to a randomized control trial by Albin et al. However, the results suggest that manual therapy may lessen aberrant resting muscle stiffness after surgical stability of the ankle and the rear foot [9].

Early rehabilitation training is crucial for healing and avoiding complications following ankle fracture surgery. Motion that is excessive or incorrect could worsen an injury by delaying recovery after ankle fracture surgery. Ni et al. developed a quantitative early passive rehabilitation training technique using finite element analysis [10]. The longitudinal interview study by Jensen et al. aims to investigate the perspectives of patients with surgically and conservatively treated ankle fractures within 10 days and six weeks after an ankle fracture. Fourteen patients were interviewed using a semi-structured interviewing plan. All patients were initially upbeat about the future, but six weeks later, many patients were still experiencing pain, and their optimism had significantly changed. Now, they had a more negative outlook on the future. Uncertainty-related feelings in patients were correlated with knowledge gaps [11].

According to Houchen-Wolloff et al., there has recently been a paradigm shift in managing Achilles tendon rupture and ankle fracture rehabilitation that emphasizes early movement. However, there is disagreement on post-operative rehabilitation for posterior/mid-foot fusion and reconstructive surgery. They examined the post-operative rehabilitation strategies of foot and ankle surgeons and associated health specialists. They concluded that there is a shortage of published research in this area and that post-operative rehabilitation for patients having this surgery differs significantly. Early rehabilitation may improve patient outcomes. However, non-unionization poses a severe risk. Additional study in a multicenter trial is required to answer this research topic [12].

In their case report, Fokmare et al. describe how they used physical therapy to manage a chronic post-operative trimalleolar ankle fracture for three weeks. The patient's general functioning improved due to the physiotherapy treatment, which comprised Mulligan's movement with mobilization, ultrasound therapy, strength training, proprioceptive training, and gait training [13].

De Ruijter et al. concluded that treating numerous extremity injuries simultaneously necessitates good pre-operative planning, considering anatomical reduction vs induced surgical damage and offering stability as required for quick rehabilitation. Physical healing is a factor in adequate rehabilitation in multi-trauma patients, but it is not the only one. Offering interdisciplinary psychosocial assistance may be crucial. Even in the early, non-weight-bearing stages of recovery, clinical rehabilitation proved immensely beneficial. It might be difficult for clinicians to keep patients thoroughly informed about their alternatives for treatment, long-term disabilities, and psychological difficulties, but it should be given priority [14].

Yoder et al.'s feasibility research shows that movement training programs for fit, seasoned runners can be modified for clinical, orthotic, and limb trauma rehabilitation settings. Additional research is required to evaluate effectiveness across a wider sample of passive dynamic ankle-foot orthosis users and injury types [15]. Compared to age-matched, healthy controls, limb salvage of type IIIB tibial fractures with segmental bone loss is linked with frequent sequelae and overall poor function. With a 24-month follow-up, the patient in the case study by Young et al. had an outstanding outcome. Despite a superficial wound infection, her latissimus flap and tibia recovered well. Her foot and ankle outcome scores for the short form-12 were slightly below average for a healthy US adult, but her short form-12 scores were above average. She is back in school and manages most everyday tasks without any problems. The outcome for our patient exemplifies the advantages of a multidisciplinary, collaborative decision-making process and emphasizes the value of limb salvage in a healthy patient [16].

The rare congenital pseudoarthrosis of the medial malleolus may result from a disruption in epiphyseal development. These could be mistaken for traumatic fractures and result in unintended surgery. To prevent the incorrect diagnosis of a fracture requiring surgery, Cerulli et al. advise performing an accurate history, an objective examination, and correlating the results of diagnostic procedures. Traumatic incidents at this location must be adequately evaluated to prevent being mistaken for malleolus fractures, which could result in overtreatment. An accurate diagnosis will result from carefully evaluating the bone boundaries and clinical instability examination. In many situations, conservative management will suffice, and the existence of pseudoarthrosis [17].

A 58-year-old man with his left leg amputated at the left talocrural joint level suffered bimalleolar fractures, a whole section of blood vessels, nerves, and tendons. He contemplated replantation surgery seven hours after the trauma was investigated by Tudoise et al. The authors combined imaging and neurophysiological testing with the clinical, functional assessment to accurately measure the postoperative circumstances throughout time. A rehabilitation program was designed to minimize unintended biomechanical changes that would restrict a person's ability to accomplish daily duties and teach the motor and sensory function of the replanted foot. The patient's protective sense, active motion angles of the left ankle joint, and muscle strength of the replanted limb gradually returned during the therapy regimen. These enable the patient to complete his daily minor housework and are sufficient for walking exercises [18].

A unique instance of a pathological bimalleolar fracture brought on by gout tophi was described by Irsay et al. in their study. This case presented a diagnostic difficulty and highlighted the value of interdisciplinary care in such circumstances. Usually, gout is not linked to an increased risk of fracture. Still, the patient had several impairments and upper and lower limb fractures, which all recovered due to the challenging rehabilitation therapy. To enhance the functional status, the patient had pharmacological treatment, physical therapy, and ankle immobilization in a knee-ankle-foot orthosis, followed by orthopedic surgical treatment for fracture fixation. Stretching, strengthening, gait and balance exercises, and soft tissue massage made up the rehabilitation program. Thermotherapy, ultrasound, electrical stimulation, and laser were additional treatment techniques utilized to lessen pain and improve the functionality of the affected joints [19].

A 30-year-old woman who had injured her right ankle from falling off a bike was the subject of a case study by Jogani et al. Since their patient had significant swelling, she was treated conservatively for eight weeks by immobilizing the lower limb with a below-the-knee splint that was not weight-bearing. When the patient was seen again, follow-up radiographs showed that the right ankle had a bimalleolar fracture. According to Budapest criteria, the diagnosis of complex regional pain syndrome was determined based on several laboratory data. This case report describes an unusual presentation of a severe form of CRPS combined with severe osteoporosis that resulted in a bimalleolar ankle fracture that was effectively treated using a multidisciplinary approach. The situation in this instance was carefully handled using an interdisciplinary approach. It strongly emphasized carefully planned rehabilitation of the injured extremity, as well as carefully regulated analgesics and cognitive psychotherapy, to break the cycle of inactivity. Regularly administered physical therapy under supervision included contrast baths, active range of motion exercises to prevent pain flare-ups, graded motor imagery, mirror therapy, and passive movements once the pain diminished [20].

## Conclusions

Ankle fractures are frequent fractures of the lower extremity. In summary, this case report highlights the effectiveness of a comprehensive rehabilitation protocol for treating Pott's fracture, which resulted in significant improvements in the patient's functional status and impairments. Future studies could explore the long-term outcomes of this rehabilitation protocol and compare its effectiveness to other treatments for Pott's fracture. The results of this study contribute to the growing body of evidence supporting the use of comprehensive rehabilitation protocols for ankle fractures. These protocols may improve patient outcomes and reduce the risk of long-term disability. The rehabilitation protocol resulted in improvements in the patient's functional status as well as the overall quality of life.
